# Case of vaginal cuff dehiscence and small bowel evisceration after laparoscopic radical cystectomy

**DOI:** 10.1002/iju5.12346

**Published:** 2021-07-13

**Authors:** Satoru Meguro, Yusuke Kirihana, Tomoyuki Kumekawa, Masato Kobayashi, Hiroshi Kameoka

**Affiliations:** ^1^ Department of Urology Hoshi General Hospital Fukushima Japan

**Keywords:** invasive bladder cancer, laparoscopic radical cystectomy, small bowel evisceration, vaginal cuff dehiscence, vaginal uterosacral ligament suspension

## Abstract

**Introduction:**

Vaginal cuff dehiscence and small bowel evisceration after laparoscopic radical cystectomy, although rare, can be a critical complication. However, little has been reported about it by urologists.

**Case presentation:**

A 79‐year‐old woman underwent laparoscopic radical cystectomy for invasive bladder carcinoma. Thirteen months postoperatively, she experienced vaginal cuff dehiscence and small bowel evisceration, and underwent emergency surgery. Intraoperatively, we detached the vaginal apex from the surrounding tissue to lengthen it and performed vaginal uterosacral ligament suspension, with no subsequent recurrence.

**Conclusion:**

Urologists should pay attention to vaginal cuff dehiscence and small bowel evisceration after laparoscopic radical cystectomy in female patients. In this case, the short vaginal length without vaginal uterosacral ligament suspension might have led to vaginal dehiscence.

**Abbreviations & Acronyms**
CT: computed tomography

**Keynote message**

Urologists should pay attention to vaginal cuff dehiscence after laparoscopic radical cystectomy in female patients, because it can lead to life‐threatening small bowel evisceration. Surgical techniques, such as conserving the length of the vaginal apex and bilateral vaginal uterosacral ligament suspension, might be important in preventing vaginal cuff dehiscence.

## Case presentation

A 79‐year‐old woman underwent laparoscopic radical cystectomy, pelvic lymph node dissection, total hysterectomy, bilateral salpingo‐oophorectomy, and ileal conduit urinary diversion for invasive bladder carcinoma at our institution. She had a history of hypertension and cerebral infarction, and her body mass index was 17.8 kg/m^2^. Intraoperatively, the urethras were completely resected both laparoscopically and extracorporeally, and the vaginal cuff was closed laparoscopically using vertically running absorbable 3‐0 polyglactin sutures. Moreover, the vaginal mucosa was closed extracorporeally using vertical, interrupted, absorbable 3‐0 polyglactin sutures. The pathological diagnosis was invasive urothelial carcinoma of the bladder with no lymph node metastasis. Although she had pain and numbness in the left leg postoperatively, which was attributed to lymph node dissection, her hospital stay was mostly uneventful and she was discharged on postoperative day 43. She did not have sexual intercourse after her discharge.

**Fig. 1:**
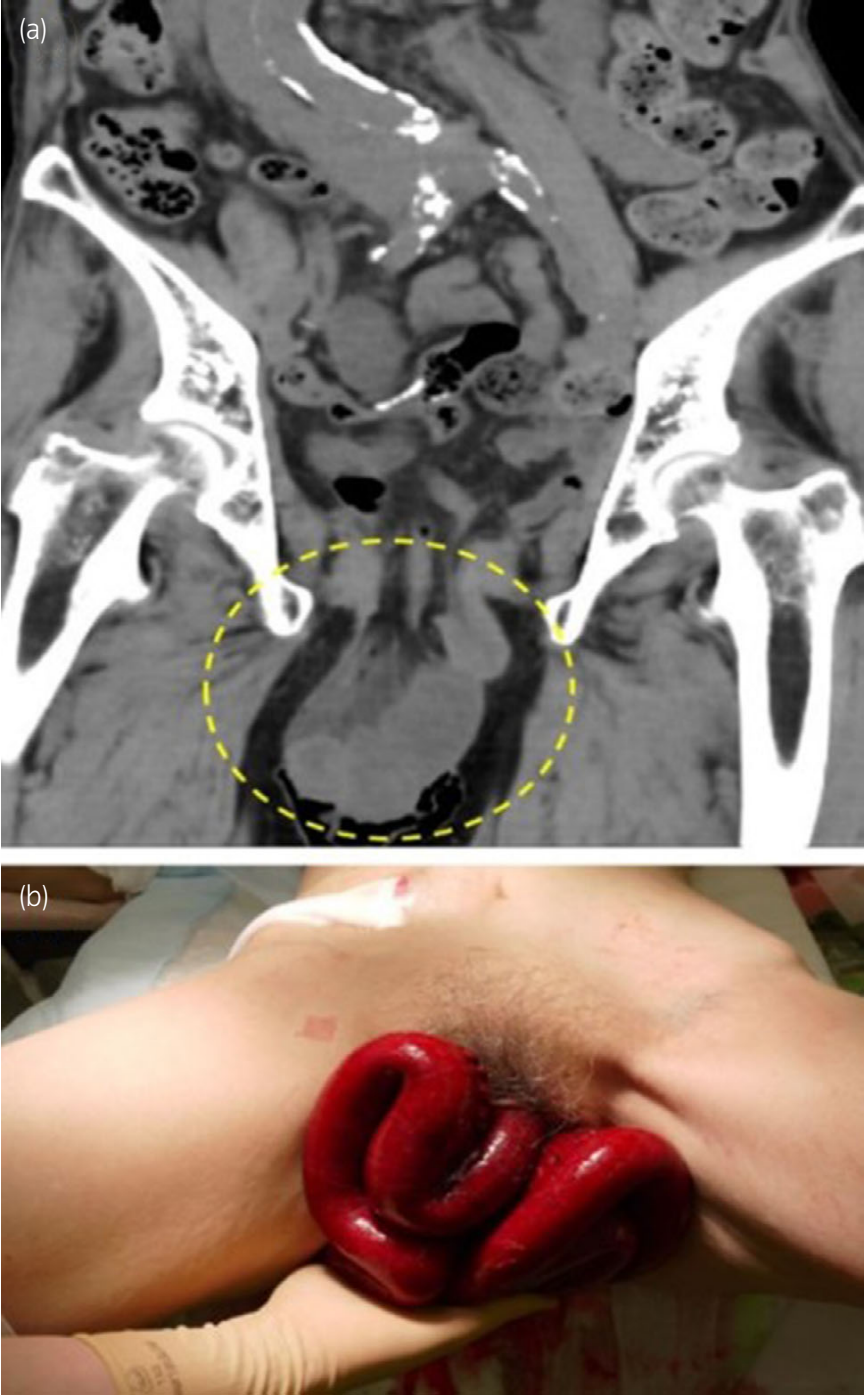
Computed tomography image of the eviscerated small bowel and gross appearance of the necrotic eviscerated small bowel. (a) The small bowel was eviscerated through the vaginal cuff. (b) The color of small bowel was reddish black in color and seemed to be necrotic.

**Fig. 2:**
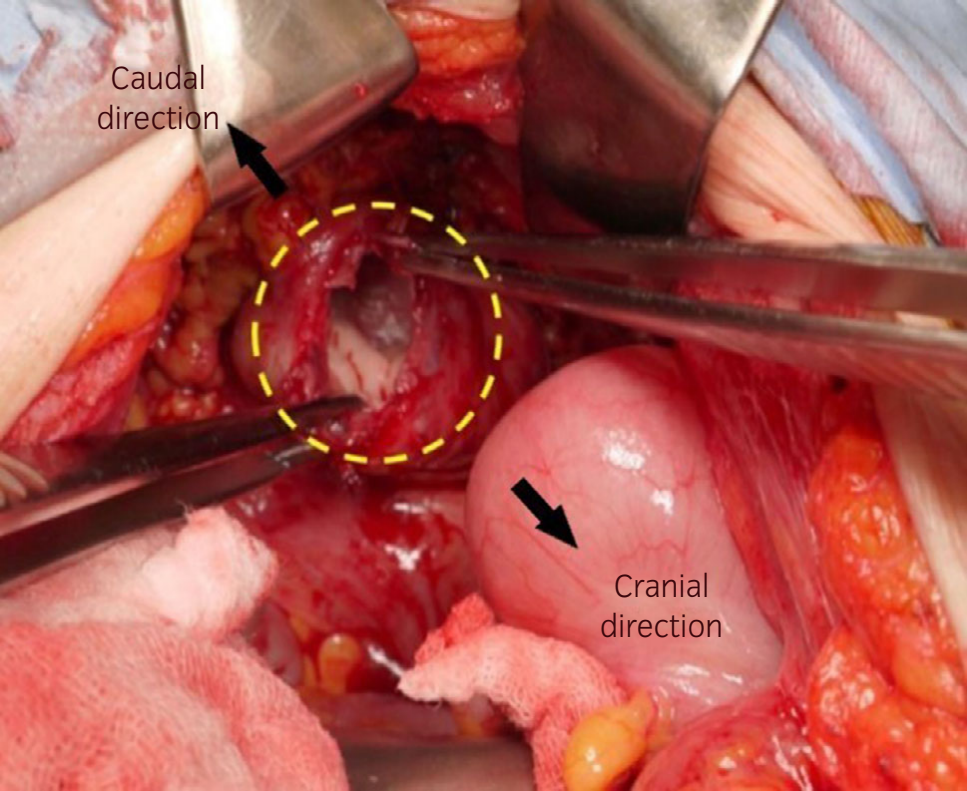
Dehisced vaginal cuff. The yellow circle shows the dehisced vaginal cuff. There was no evidence of necrotic tissue in the vaginal cuff. The black arrows indicate the cranial and caudal directions.

**Fig. 3:**
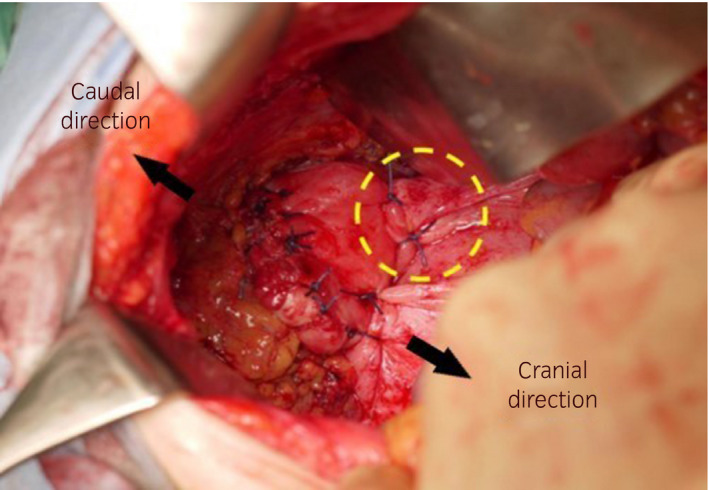
Repair of the dehisced vaginal cuff. The dehisced vaginal cuff was repaired by simple interrupted vertical sutures with absorbable polydioxanone 0 sutures. The vaginal cuff was attached to the right uterosacral ligament, creating the vaginal uterosacral ligament suspension (yellow circle). The attached left uterosacral ligament is behind the vaginal cuff in this figure. The black arrows indicate the cranial and caudal directions.

Thirteen months postoperatively, she presented at our institution with the complaints of lower abdominal pain and nausea. Physical examination and CT revealed prolapse of the small bowel through the vagina (Fig. 1a). The small bowel was reddish black in color and seemed to be necrotic (Fig. 1b). Although her vital signs were normal, we considered that she needed emergency surgery. The eviscerated small bowel was resected outside the body and replaced intraperitoneally through the vagina, and the resection stumps were anastomosed by general surgeons using an automatic anastomotic device. Since there was no evidence of necrotic tissue in the vaginal cuff, we detached the vaginal apex from the surrounding tissue and repaired the vaginal cuff by simple interrupted vertical sutures. Additionally, we attached the uterosacral ligaments bilaterally to near the vaginal apex for vault suspension using absorbable polydioxanone 0 (Figs 2 and 3). Perineoplasty was additionally performed.

**Fig. 4:**
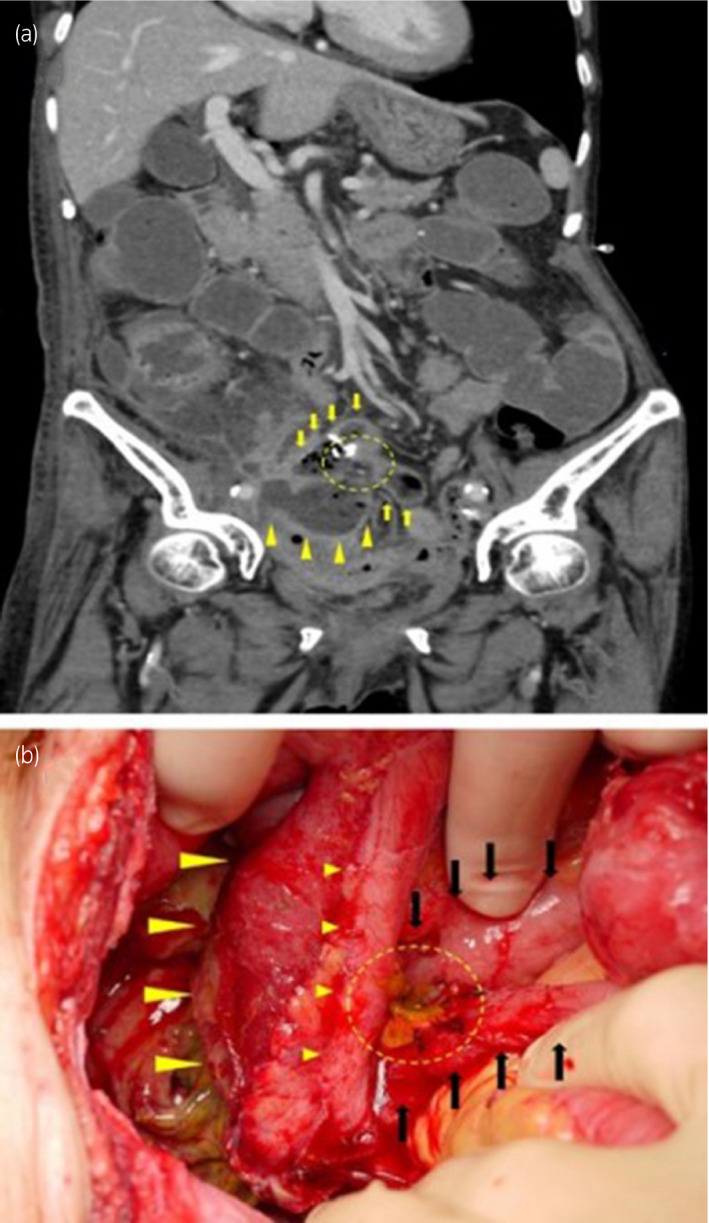
Computed tomography showing anastomotic leakage and an intraperitoneal abscess following a fistula of the ileal conduit, and intraoperative visualization of the anastomotic leakage seen. (a) The yellow arrowheads and the yellow arrows indicate the ileal conduit and anastomosed stumps of the resected small bowel, respectively. The yellow circle shows anastomotic leakage. (b) The big yellow arrowheads and the small yellow arrowheads indicate the ileal conduit and ureter, respectively. The black arrows indicate the anastomosed stumps of the resected small bowel. Anastomotic leakage and stool leakage were recognized (yellow circle).

Ten days after the surgical repair, serum markers of inflammation were elevated and stool was recognized exiting from the urostomy. CT revealed anastomotic leakage and an intraperitoneal abscess following a fistula of the ileal conduit (Fig. 4a). A third surgery was emergently performed, which confirmed the CT findings (Fig. 4b). During the surgery, the ileal conduit was removed and bilateral nephrostomies were constructed. The segment of the small bowel with anastomotic leakage was resected and the oral stump of the bowel was anastomosed with the cecum by the general surgeons.

After the third surgery, the patient’s inflammatory markers decreased gradually and she slowly recovered. After an additional rehabilitation period, she was discharged 49 days after the third surgery. Follow‐up 4 months after her discharge indicated that her outpatient course was uneventful, without any signs of recurrence.

## Discussion

Vaginal cuff dehiscence after total hysterectomy is a rare but serious complication that can be life‐threatening if it leads to small bowel evisceration.^1^ Previous studies have variously reported the incidence of vaginal cuff dehiscence after total laparoscopic hysterectomy and total abdominal hysterectomy of 1.1 to 4.9% and 0.12%, respectively,^2^, ^3^ suggesting that total laparoscopic hysterectomy carries the risk of vaginal cuff dehiscence.^4^ However, the incidence of vaginal cuff dehiscence after total laparoscopic hysterectomy following laparoscopic radical cystectomy might not be the same as that with total laparoscopic hysterectomy alone, because the anterior vaginal wall is often defected during laparoscopic radical cystectomy when the urethra is resected. A Japanese multicenter cohort study reported that 7 of 100 female patients underwent emergency surgery for bowel evisceration as a result of vaginal cuff dehiscence after laparoscopic radical cystectomy.^5^ Moreover, several cases of transvaginal bowel evisceration after robot‐assisted radical cystectomy or open pelvic exenteration have also been previously reported.^6^, ^7^, ^8^ However, there have not been too many reports about vaginal cuff dehiscence after laparoscopic radical cystectomy.

There are several risk factors for vaginal cuff dehiscence. Hematoma, pelvic floor defects, poor wound healing, infection, postoperative vaginal trauma such as due to intercourse, age, smoking, prior radiation therapy, increased intraabdominal pressure, chronic steroid administration, and malnutrition have all been reported as possible risk factors.^2^, ^9^, ^10^, ^11^, ^12^ Several methods have been reported as surgical techniques to prevent vaginal dehiscence, including bilateral vaginal uterosacral ligament suspension, vertical suturing, conserving the length of the vaginal apex, and cutting with minimal coagulation.^13^, ^14^

In the first surgery in our patient, while vertical suturing and cutting with minimal coagulation were performed, vaginal uterosacral ligament suspension was not performed and the vaginal length was extremely short because of the proximity of the carcinoma to the urethra, since we had to perform as wide a tissue resection as possible so as not to leave any residual carcinoma. In the second surgery, we detached the vaginal apex from the surrounding tissue to lengthen it and also performed vaginal uterosacral ligament suspension, with no subsequent signs of recurrence. Generally, a shorter vagina is associated with cuff dehiscence with postoperative sexual intercourse.^15^ In this case, however, the patient did not have sexual intercourse. We opined that a short to negligible vaginal length leads to exertion of tension on the vaginal cuff. In the absence of vaginal uterosacral ligament suspension, the sutured cuff might not be able to withstand increased abdominal pressure. Although a drawback of vaginal uterosacral ligament suspension is the risk of ureteral kinking,^13^ this is not of concern in radical cystectomy because the ureters bilaterally are clearly identified during surgery. Therefore, vaginal uterosacral ligament suspension, in particular, might be a good method for preventing vaginal cuff dehiscence in total hysterectomy following radical cystectomy. However, further research is needed to determine the incidence and factors or mechanisms of vaginal cuff dehiscence after laparoscopic radical cystectomy.

We report a case of vaginal cuff dehiscence and transvaginal small bowel evisceration after laparoscopic radical cystectomy. In this case, the short vaginal length without vaginal uterosacral ligament suspension might have led to vaginal dehiscence. Vaginal uterosacral ligament suspension, in particular, might be an effective method for preventing vaginal cuff dehiscence after total hysterectomy associated with radical cystectomy, without the risk of ureteral kinking. Urologists should pay attention to vaginal cuff dehiscence and small bowel evisceration after laparoscopic radical cystectomy in female patients, although these complications are only rarely experienced by urologists.

## Conflict of interest

The authors declare no conflict of interest.

## Approval of the research protocol by an institutional reviewer board

In this research the protpcol apprived by a reviewer board in our institution was not applicable.

## Informed consent

Informed consent was obtained from the patient for the publication of this article and accompanying the images.

## Registry and the registration no. of the study/trial

A registration of this study as a clinical trial in our institution was not applicable.
